# On the Therapeutic Value of Nux Vomica in the Treatment of Asthenic Forms of Disease

**Published:** 1865-04

**Authors:** A. Middleditch

**Affiliations:** Waterloo, Iowa


### ON THE THERAPEUTIC VALUE OF NUX VOMICA IN THE TREATMENT OF ASTHENIC FORMS OF DISEASE.

By A. MIDDLEDITCH, M. D., Waterloo, Iowa.

About four years ago, I had under treatment a case of dysentery, attended by a low, slow form of fever, which, under the use of tonics and other treatment usually followed in such cases, the patient, a child about two years old, became daily worse, until it sunk into a state of extreme exhaustion, with coma and a cadaverous appearance. At this period, I asked Dr. B. to see the case with me. In his opinion, the last hope had nearly expired, and treatment useless; and I was left to act from my own resources. Prescribed Nux Vomica in doses of 1-60 gr. of the solid extract in aqueous solution every hour. After a few doses, I could begin to perceive a little change; the pulse beat fuller and slower; child less stupid, and the face less death-like. These symptoms all gradually improved and the patient recovered.

Soon after this, I was called to consult with the attending physician, in the case of a young man in the last stages of Typhoid fever. His immediately dangerous symptom was death from coma, and which was already so intense that it was impossible to arouse him to the least consciousness. This young man had been very sick for more than two weeks, and it was very evident to my mind that this stupor, which had gradually supervened, was caused not by oppression, but by prostration of the nervous centres; and, therefore, instead of blistering, the question arose, is there not *something* which will *create* nerve force, rather than arouse an unnatural power for a few hours only, by blisters? I believed that Nux Vomica would do this, if anything could; which we gave in doses of 1-12 gr., every hour, of the solid extract. Very soon we saw that that expiring flame was being retouched with the brain's own native fire; those eyes, which a few hours before were almost in rigor mortis, began to awaken as if touched by a magic wand, and we had the satisfaction of seeing this patient recover.

The above are two examples of cases, among many, where I have used Nux with great advantage. In every disease, whatever the name, when there was reason to suppose that the nervous centres have been overcome and prostrated, indicated by coma, tremors, etc., the employment of this remedy has been attended not by any means with invariable success, but with great utility in many cases; and should I be asked with what remedy I have been most highly pleased—what have I used which has given me the greatest assurance, has disarmed the angel of death and cheated him of his victory? —I should answer, Nux Vomica.

I am anxious, therefore, to humbly submit it to the test of practitioners, in the exhaustion and prostration of the nervous centres, believing that there is no other article of the Materia Medica so potent for arousing and revivifying their lost power.

March 4th, 1865.

